# Supplementary material for: Comparative readability of information on different treatment options for breast cancer, based on WeChat public accounts
Source: PLoS One. 2025 Jan 24;20(1):e0317032. doi: 10.1371/journal.pone.0317032 (PMC11759364; doi:10.1371/journal.pone.0317032)
Supplement: S2 File — (DOCX) [file pone.0317032.s002.docx]

**The Chinese version of the Suitability Assessment of Materials (SAM)**

Xianwen Li

| 1. **Content Dimension** |
| --- |
| (a) Purpose is evident  2 Purpose is explicitly stated in title, or cover illustration, or introduction  1 Purpose is not explicitly. It is implied, or multiple purposes are stated  0 No purpose is stated in the title cover illustration, or introduction |
| (b) Content about behaviors  2 Thrust of the material is application of knowledge/skills aimed at  1 Desirable reader behavior rather than non-behavior facts  0 Nearly all topics are focused on non-behavior facts |
| (c) Scope is limited  2 Scope is limited to essential information directly related to the purpose. Experience shows it can be learned in time allowed.  1 Scope is expanded beyond the purpose; no more than 40 percent is non-essential information. Key reports can be learned in time allowed  0 Scope is far out of proportion to the purpose and time allowed |
| (d) Summary or review included  2 A summary is included and retells the key messages in different words and examples  1 Some key ideas are reviewed.  0 No summary or review is included |
| 1. **Literacy Demand Dimension** |
| (a) Writing style, active voice  2 Both factors:  (1) Mostly conversational style and active voice  (2) Simple sentences are used extensively; few sentences contain embedded information  1 Both factors:  (1) About 50 percent of the text uses conversational style and active voice  (2) Less than half the sentences have embedded information  0 Both factors:  (1) Passive voice throughout  (2) Over half the sentences have extensive embedded information |
| (b) Vocabulary uses common words  2 All three factors:  (1) Common words are used nearly all of the time  (2) Technical, concept, category, value judgment (CCVJ) words are explained by examples  (3) Imagery words are used as appropriate for content  1 Both factors  (1) Common words are frequently used  (2) Technical and CCVJ words are sometimes explained by examples  0 Two or more factors:  (1) Uncommon words are frequently used in lieu of common words  (2) No examples are given for technical and CCVJ words  (3) Extensive jargon |
| (c) Context is given first  2 Consistently provides context before presenting new information  1 Provides context before new information about 50 percent of the time  0 Context is provided last or no context is provided |
| (d) Learning aids via “road signs,” subtitles and captions  2 Nearly all topics are preceded by an advance organizer (a statement that tells what is coming next)  1 About 50 percent of the topics are preceded by advance organizers  0 Few or no advance organizers are used |
| 1. **Graphics** |
| (a) Cover graphic shows purpose  2 The cover graphic is:  (1) friendly  (2) attracts attention  (3) clearly portrays the purpose of the material to the intended audience  1 The cover graphic has one or two of the superior criteria  0 The cover graphic has none of the superior criteria |
| (b) Type of graphics  2 Both factors:  (1) Simple, adult-appropriate, line drawings/sketches are used  (2) Illustrations are likely to be familiar to the viewers  1 One of the superior factors is missing  0 None of the superior factors are present |
| (c) Relevance of illustrations  2 Illustrations present key messages visually so the reader/viewer can grasp the key ideas from illustrations alone. No distractions  1 (1) Illustrations include some distractions  (2) Insufficient use of illustrations  0 One factor:  (1) Confusing or technical illustrations (non-behavior related)  (2) No illustrations, or an overload of illustrations |
| (d) Lists and tables explained  2 Step-by-step directions, with an example, are provided that will build comprehension  and self-efficacy  1 “How-to” directions are too brief for reader to understand and use the graphic without  additional counseling  0 Graphics are presented without explanation |
| (e) Captions used for graphics  2 Explanatory captions with all or nearly all illustrations and graphics  1 Brief captions used for some illustrations and graphics  0 No captions |
| **4. Layout and typography** |
| (a) Layout factors  2 At least 5 of the following 8 factors are present:  • Illustrations are on the same page adjacent to the related text  • Layout and sequence of information are consistent, making it easy for the patient to predict the flow of information  • Visual cuing devices (shading, boxes, and arrows) are used to direct attention to specific points or key content  • Adequate white space is used to reduce appearance of clutter  • Use of color supports and is not distracting to the message. Viewers need not learn color codes to understand and use the message  • Line length is 30-50 characters and spaces  • There is high contrast between type and paper  • Paper has non-gloss or low-gloss surface  1 At least three of the superior factors are present  0 (1) Two (or less) of the superior factors are present  (2) Looks uninviting or discouragingly hard to read |
| (b) Typography  2 The following 4 factors are present:  • Text type is in uppercase and lowercase serif (best) or sans-serif  • Type size is at least 12 point  • Typographic cues (bold, size, color) emphasize key points  • No ALL CAPS for long headers or text  1 Two of the superior factors are present  0 One or none of the superior factors are present or six or more type styles and sizes are used on a page |
| (c) Subheads (“chunking’) used  2 (1) Lists are grouped under descriptive subheadings or “chunks”  (2) No more than five items are presented without a subheading  1 No more than seven items are presented without a subheading  0 More than seven items are presented without a subheading |
| **5. Learning stimulation and motivation** |
| (a) Interaction used  2 Problems or questions presented for reader responses  1 Question-and-answer format used to discuss problems and solutions (passive interaction)  0 No interactive learning stimulation provided |
| (b) Behaviors are modeled and specific  2 Instruction models specific behaviors or skills (for example, for nutrition instruction, emphasis is given to changes in eating patterns or shopping or food preparation/cooking tips; tips to read labels)  1 Information is a mix of technical and common language that the reader may not easily interpret (e.g., technical: starches – 80 calories per serving; high fiber – 1 to 4 grams of fiber in a serving)  0 Information is presented in nonspecific or category terms such as the food groups |
| (c) Motivation, self-efficacy  2 Complex topics are subdivided into small parts so that readers may experience small successes in understanding or problem-solving, leading to self-efficacy  1 Some topics are subdivided to improve the readers’ self-efficacy  0 No partitioning is provided to create opportunities for small successes |
| **6. Cultural appropriateness** |
| (a) Match in logic, language, experience (LLE)  2 Central concepts/ideas of the material appear to be culturally similar to the LLE of the target culture  1 Significant match in LLE for 50 percent of the central concepts  0 Clearly a cultural mismatch in LLE |
| (b) Cultural image and examples  2 Images and examples present the culture in positive ways  1 Neutral presentation of cultural images or foods  0 Negative image such as exaggerated or caricatured cultural characteristics, actions, or examples |
| **Total SAM score:**  • The maximum possible total score is 42 points – 100%  • The dimension and total scores of the scale were calculated as a score rate (material score/total score × 100%)  • Interpretation of SAM percentage ratings:  70-100 percent superior material  40-69 percent adequate material  0-39 percent not suitable material |

Scoring: 2 points for superior rating, 1 point for adequate rating, 0 points for not suitable rating, N/A if the factor does not apply to this material
