# Supplementary material for: Comparative readability of information on different treatment options for breast cancer, based on WeChat public accounts
Source: PLoS One. 2025 Jan 24;20(1):e0317032. doi: 10.1371/journal.pone.0317032 (PMC11759364; doi:10.1371/journal.pone.0317032)
Supplement: Attachment — Submitted filename: Response to reviewers.docx [file pone.0317032.s004.docx]

**Point-by-point response to editor and reviewer**

Dear editor and reviewer,

Thank you for your time and effort in reviewing our manuscript, and the constructive comments which help us improve the quality of this manuscript. We appreciate the positive comments on our study, **and we have revised our manuscript to address each comment and concern, with the corresponding modifications in the revised manuscript marked in blue.** The detailed responses to all comments are as follows.

Sincerely,

Bingyan Li

Corresponding author:

Lunfang Xie

E-mail: [527548725@qq.com](mailto:527548725@qq.com)

**Journal Requirements:**

1. Please ensure that your manuscript meets PLOS ONE's style requirements, including those for file naming.

**Response:** We have modificated the manuscript and file names to meet PLOS ONE's style requirements including font, font size, line spacing, fig , etc.

2. In your Methods section, please include additional information about your dataset and ensure that you have included a statement specifying whether the collection and analysis method complied with the terms and conditions for the source of the data. Please also clarify if the data collection method involved, or was at risk of, collecting personal health information.

**Response:** Thank you for the suggestion. In the methods section, we have added a detailed process for screening WPAs and articles, as described in page 6, line 121-126 and page 7, line 128-136. Basic characteristics information extraction for articles, as described in page 7, line 143-151, and the detailed dataset is also presented as S1 File in the supporting information. The 136 articles included in this study did not involve personal health information and are clarified in the text, as described in page 7, line 136-137.

3. In your Data Availability statement, you have not specified where the minimal data set underlying the results described in your manuscript can be found. PLOS defines a study's minimal data set as the underlying data used to reach the conclusions drawn in the manuscript and any additional data required to replicate the reported study findings in their entirety. All PLOS journals require that the minimal data set be made fully available.

**Response:** We gratefully appreciate for your comment. The “Data Availability Statement” have listed before the acknowledgments. Please see the revised content on page 20, line 414-415. The minimal dataset for our study is the readability score of 136 articles, which has been added as the S1 File in the revised supporting information. Please see the revised content on page 26, line 538-541. Thanks again for your valuable comment.

Special thanks to your constructive comments.

**Responses to Reviewer #1**

1: This is an interesting study and I congratulate the authors on this manuscript. It is interesting to note the difference in CAM and non-CAM online information.

**Response:** We thank you for your appreciation of our efforts. Your comments and advices are of great help to improve our study and manuscript. We have revised the article and made the following modifications based on your comments.

1) Page 9–end of the page it reads “in worldwide, Approximately 29-57%”–could this be changed to “Worldwide, approximately 29-57%”.

**Response:** Thank you for pointing out this problem in manuscript. we have made the revision on page 4, line 79.

2) Introduction is very thorough, but this information can be presented more succinctly.

**Response:** Thank you for the detailed review. We have merged and made concise the two paragraphs describing WPAs in the Introduction. Please see the revised content on page 4, line 72-78.

3) First paragraph of discussion seems to be repeating what has already been discussed in "Introduction".

**Response:** Thank you for pointing this out. We have modified the first paragraph of discussion. Please see the revised content on page 14, line 270-276.

4) The discussion is very long. I would recommend shortening the length of the discussion to make it easier to read and follow.

**Response:** Thank you for your suggestion. We reorganized the discussion section, removing duplicates and highlighting the shortcomings and suggestions for improvement of the readability of different treatment options.

Once again, we appreciate you for your time and effort on revision, and the constructive comments and advices that improve out manuscript.

If the reviewers and editors have questions or are not satisfied with our revisions and responses, please let us know. We are willing to actively modify the text to improve the quality of the article.
