# Supplementary material for: Comparative readability of information on different treatment options for breast cancer, based on WeChat public accounts
Source: PLoS One. 2025 Jan 24;20(1):e0317032. doi: 10.1371/journal.pone.0317032 (PMC11759364; doi:10.1371/journal.pone.0317032)
Supplement: Attachment — Submitted filename: renamed_76eb1.docx [file pone.0317032.s005.docx]

**Point-by-point response to reviewers**

Manuscript ID PONE-D-23-12580R1

Title: Comparative readability of information on different treatment options for breast cancer, based on WeChat public accounts

Dear editors and reviewers,

We appreciate the time and effort that you and the reviewer dedicated to our manuscript. We are also grateful for the insightful comments and valuable suggestions. In the revised manuscript, the changes are marked in the “Track Changes” mode. Below are notes on the revised manuscript, with our answers highlighted in blue. We hope that the revision has addressed the concerns raised by the reviewer, and the revision is now found satisfactory. We are ready to work on resolving any remaining issues.

Sincerely,

Bingyan Li

Corresponding author:

Lunfang Xie

E-mail: [527548725@qq.com](mailto:527548725@qq.com)

**Responses to Reviewer #2**

Was the readability assessment performed by two physicians? Since this is a general audience article, why not ask a non-medical person to evaluate it? Would you get a different result?

**Response:** We gratefully appreciate your comments.

Q1: Was the readability assessment performed by two physicians?

A: No. In our study, two postgraduate medical students (BL and WY) used SAM to evaluate the readability of the articles. BL and WY are part of our research team who has been studying on the readability of health information in China in recent years. They are proficient in the use of SAM and have published six articles as belows.

[1] **Yang W**, **Li B**, **Xie L**, et al. Quality evaluation of health information about breast cancer treatment found on WeChat public accounts. Arch Public Health. 2023;81(1):170. Published 2023 Sep 15.

[2] Wang Q, **Xie L**, Xing L, et al. Development of the readability evaluation index system for online health information. Chinese Journal of Nursing Education 2022;19(01):14-20.

[3] Wang L, **Xie L**, Wang Q, et al. Readability assessment of online health information for Systemic Lupus Erythematosus. Nursing Research of China 2020;34(24):4344-4349.

[4] Wang L, **Xie L**, Wang Q, et al. Study Status and Prospect of Readability Assessment of Online Health Information. Journal of Medical Informatics 2020;41(12):20-25+40.

[5] ZhiWei W, **Xie L**, YuanYuan F, et al. The readability assessment of printed education materials on Hypertension. Chinese Journal of Health Education 2020;36(12):1122-1125+1167.

[6]. Wang Q, **Xie L**, Li M, et al. Review on readability assessment tools for health education materials. Chinese Journal of Health Education 2019;35:66-71.

[7]. Wang Q, **Xie L**, Li M, et al. Evaluation of quality and readability of health education materials in Anhui Province from nurses’perspectives. Chinese Journal of Modern Nursing 2019;25:1093-98.

Please see the revised content on page 9, line 186-187.

Q2: Why not ask a non-medical person to evaluate it? Would you get a different result?

A: The SAM was developed to be used by professionals, not by a non-medical person. According to the literature review, most study on readability assessment of medical information chose medical specialists and medical students as evaluators, because they are more familiar with medical knowledge, they can evaluate the readability based on the accuracy of medical information. If we ask a non-medical person to evaluate, the result could be very different, but not credible.

**Responses to Reviewer #3**

The researchers selected 136 articles on various aspects of treatment for breast cancer that were published on 37 different WeChat accounts. Readability is an important to help ensure equity of care irrespective of levels of formal educations.

I appreciate the work that has gone into the paper, including responding to previous review comments.

Unfortunately I had difficulty in understanding the methods and the rationale for the study which lacked many important details and also lacked the source data that I would expect in a such a study, even allowing for the language difficulties.

**About Data access:**

1. Although the researchers say they have provided all relevant material, they only provide the readability scores for these papers and not the papers themselves.

Even though I do not understand Chinese, the researchers have used a tool to evaluate readability that focuses on the visual layout of material, including the use of headings, space, illustrations and other graphics (top of page 8: "graphics, layout and typography, learning stimulation and motivation, and cultural appropriateness”) . These are actually easy to understand irrespective of language, as the analysis or the paper explains. Currently, the results of the study are presented - ie the readability score - but not the actual examples that the study is based on. If this study was about readability of resources produced in English, I would want to look at these original papers which would immediately tell me about the range of resources. This option to see the source material should be available to Chinese readers.

The most important aspect of readability for resources in English is the language used, the structure of the language and the simplicity of the words. At it’s simplest, this involves using short simple words, short sentences and short paragraphs, using the Flecsh-Kincaid or similar reading ease score. Because structural challenges of the Chinese language have not led to a translatable version of Flecsh-Kincaid or similar, test readability has been dropped from the analysis, only relying on visual aspects mentioned above, making access to the source resources essential.

**Response:** Thanks for bringing up the concern. The basic characterization information for 37 WPAs and 136 articles is listed in S1 file, which is also added in Supporting Information on page 28, lines 579-581. The S1 File supplements the Chinese titles, WPA owners, links to the original articles and treatment options for all the WPAs and articles, so that experts and readers can access to the source resources.

**About Background and general comments:**

1. As an English reader, and as the publication is in English, it is not helpful that the paper has no explanation for WeChat as a publisher. Without this context it is difficult to decide on the context of this information. WeChat appears to be similar to FaceBook, and so-called Official or Public accounts look similar to a Facebook profile for individuals where individuals or organisations can publish content commercial or free content, also building up followers. Content doesn’t appear to need to pass any quality control but is just self-published.

**Response:** Based on your suggestions, we have added more background information about WeChat, such as the developers and use of WeChat on page 4, line 73-76, the WPA owners on page 4, line 76-78, and the description of WeChat's qualification of WPA owners and content quality control is on page 5, line 99-104.

2. WeChat would be easily known to a Chinese reader - with apparently 360 million users in 2021 according to the paper - so will be a vast source of potential information, but there is little information for how the researcher selected 136 articles or the 37 WeChat profiles. The last paragraph on page 6 just says ’37 WPAs were selected’.

**Response:** We gratefully appreciate your comments. We have added more detailed information for how the researcher selected 136 articles or the 37 WeChat profiles, such as the search process for WPAs, the inclusion and exclusion criteria for articles and WPAs, and final compliance with the criteria. Please see the revised content on Page 6, Line 126-134 and Page 7, Line 137-146.

3. Without, for example, knowing if these are just personal accounts or breast cancer charities/NGOs I have no interest in readability scores.

**Response:** Thank you for your constructive comment. We've added WPA owners content on page 8, line 156-158 and page 10, line 211-212. The information for 37 WPA owners is listed in S1 file in Supporting Information on page 28, lines 579-581.

4. The cut-off criteria for poor readability also seems like an unusually low threshold - ie anything above reading grade 6. Very little general medical information in English achieves reading grade 6 or lower, and yet the main result from the paper is that much of the material they looked at is reading grade 7.

**Response:** Grade 6 is calculated based on the formula, which mainly calculates the difficulty level of word comprehension from the perspective of textual factor, while the readability evaluation in this study is based on six dimensions of SAM from the perspective of non-textual factors. So “adequate” in our study might not be equal to grade 7.

5. Although ‘content’ of the resources is included in the SAM algorithm, the paper doesn’t explain how this was evaulated - unless I missed this, and apologise. Yet content is arguably the most important criteria for knowing whether the material is a good source of information. I don’t want to know whether unreliable information is easy to read.

**Response:**

(1) The content dimension in the SAM consists of 4 factors, as described in the following table.

**Content Dimension**

(a) Purpose is evident

2 Purpose is explicitly stated in title, or cover illustration, or introduction

1 Purpose is not explicitly. It is implied, or multiple purposes are stated

0 No purpose is stated in the title cover illustration, or introduction

(b) Content about behaviors

2 Thrust of the material is application of knowledge/skills aimed at

1 Desirable reader behavior rather than non-behavior facts

0 Nearly all topics are focused on non-behavior facts

(c) Scope is limited

2 Scope is limited to essential information directly related to the purpose. Experience shows it can be learned in time allowed.

1 Scope is expanded beyond the purpose; no more than 40 percent is non-essential information. Key reports can be learned in time allowed

0 Scope is far out of proportion to the purpose and time allowed

(d) Summary or review included

2 A summary is included and retells the key messages in different words and examples

1 Some key ideas are reviewed.

0 No summary or review is included

The evaluation process for the content dimension is as same as that for the other dimensions. We have added relevant content on improving inter-rater consistency and the assessment process. Please see the revised content on page 9, line 186-194.

(2) This article is part of our study named ‘quality and readability evaluation on breast cancer treatment information on WPAs’, and anther paper about the quality of informaition has been published with findings of **moderate quality**, cf. [33]. In particular, DISCERN which consists of three parts and 16 items was used to assess quality of 136 articles. **The first part (Items 1–8) is related to the reliability of the article; the second part (Items 9–15) evaluates the specific details of treatment choice information;** the third part (Item 16) describes the overall quality of the source publication of health information on treatment choices. **The reliability dimension was assessed as fair**, with criteria including: clear information sources, explicit objectives, relevant content themes, objective content, and mention of uncertainties. Please see the revised content on page 6, line 120-122.

**About Introduction:**

1. Although breast cancer clearly affects a very large numbers of people I worry when I read sentences like:

"Worldwide, 29–57% of breast cancer patients have searched for relevant health information online.” (halfway down page 4).

The context is the rapid development of the internet and this statement is based on two tiny studies (approx 200 people) that were published over a decade ago.

**Response:**Thank you for the detailed review. The statement you mentioned has been updated with new references. Please see the revised content on page 4, line 72-73.

2. I have no confidence that 66.7% of people with breast cancer in China searched for information online. It is not true and isn’t a fact. It makes be see this paper, unfortunately, as a lot of generated data, that is disconnected from any real-world understanding of the issues related to readability of real information used by real people who depend on this for essential life choices about their future.

**Response:**Thank you for your suggestion. We have rephrased the use of WPAs in breast cancer patients, as well as added the sources from which this conclusion was reached to add credibility, and added some literatures to support the data. See revisions to lines 83-86 on page 4.

**About Statistics:**

1. Perhaps this is a cultural difference that I am not aware of, but given the paper refers to an unequal distribution of data, it is appropriate to present median and IQR for all data. I don’t understand why the IQR is given as a numeral rather than a range - ie being presented as an SD when standard practice ot to give a clear interquartile range.

**Response:** In our understanding, the IQR can be either a range or the difference between P75 and P25, cf. [36]. Now, we have expressed IQR as a range based on your suggestion. See Table 1 on page 11, Table 2 on page 13, and Table 3 on page 14 for revisions, and the representation of IQR in the text has also been revised (see Page 2, Line 28-33 and Page 11, Line 222).

Once again, we appreciate you for your time and effort on revision, and the constructive comments and advice that improve out manuscript.

If the reviewers and editors have questions or are not satisfied with our revisions and responses, please let us know. We are willing to actively modify the text to improve the quality of the article.
