# Supplementary material for: Comparative readability of information on different treatment options for breast cancer, based on WeChat public accounts
Source: PLoS One. 2025 Jan 24;20(1):e0317032. doi: 10.1371/journal.pone.0317032 (PMC11759364; doi:10.1371/journal.pone.0317032)
Supplement: Attachment — Submitted filename: Response to reviewers.docx [file pone.0317032.s006.docx]

**Point-by-point response to reviewer**

Manuscript ID PONE-D-23-12580R1

Title: Comparative readability of information on different treatment options for breast cancer, based on WeChat public accounts

Dear editors and reviewers,

We appreciate the time and effort that you and the reviewer dedicated to our manuscript. We are also grateful for the insightful comments and valuable suggestions. In the revised manuscript, the changes are marked in the “Track Changes” mode. Below are notes on the revised manuscript, with our answers highlighted in blue. We hope that the revision has addressed the concerns raised by the reviewer, and the revision is now found satisfactory. We are ready to work on resolving any remaining issues.

Sincerely,

Bingyan Li

Corresponding author:

Lunfang Xie

E-mail: [527548725@qq.com](mailto:527548725@qq.com)

**Responses to Reviewer #4**

This is a fascinating study exploring a tool of which I was unaware. WeChat and its WPAs seem like a popular resource for patients seeking reliable medical information, and I applaud your recommendations for making them easier to use and understand.I do have a few questions, though, about your methods.

**About Data access:**

1. In Section 2.2.1 you mention that a translation of the Suitability Assessment of Materials (SAM) was used to gauge the readability of each article. As you note the SAM recommends using the Flesch-Kincaid readability formula to assess the Literacy Demand of an article, and you also note that this particular tool has not been translated into Chinese and therefore this component was deleted. How, then, was the Literacy Demand calculated? We see a score for this component in each of the Tables, but I’m unsure how this was determined. Did you use the original English version of Flesch-Kincaid, or did you substitute something else? Did you use the Chinese Readability Index Explorer mentioned earlier in the Introduction? I re-read the paper a few times and this escaped me, and I apologize if I simply missed it.

**Response:** We gratefully appreciate your comments.

Q1: How was the Literacy Demand calculated ? Did you use the original English version of Flesch-Kincaid, or did you substitute something else? Did you use the Chinese Readability Index Explorer mentioned earlier in the Introduction?

A: We did not use the original English version of Flesch-Kincaid or other substitute tool. We did not use the Chinese Readability Index Explorer either. In our study, the SAM referred to the Chinese translation by Chinese scholar Xianwen Li in 2013 was used. “Literacy Demand Dimension” in the English version has five items including Reading grade level, Writing style, Vocabulary uses common words, Context is given first, and Learning aids. Reading grade level was assessed by the Flesch-Kincaid readability formula. But this item was not in the Chinese version of the SAM. So we assessed the literacy demand with the rest 4 items. In order to clarify this issue, the article had been revised accordingly. See page 8, line 172-177 and page 9, line 180-185. The dimension and item details of the Chinese version of the Suitability Assessment of Materials (SAM), which had been added as the S2 File in the revised supporting information. Please see the revised content on page 28, line 591-592. The changes in the current article are also reflected, see page 9, line 188-189.

2. As has been noted by other Reviewers, the Discussion section is too long, and parts of it seem speculative as opposed to rooted in fact. For example, on Page 18, you discuss the benefits of using graphics to support concepts discussed in the text, and you provide a relevant citation. This is well done. However, on Page 19, you mention that patients’ interest level in CAM articles is due to including the topic of nutritional therapy and mentioning “commonly used foods, such as oranges and spinach”. Where is the citation for that? How did you determine interest? Does WeChat display the number of reads for a WPA? Even if you had such usage data, how can you determine which aspect of the paper engendered interest? Does WeChat allow users to post comments, and, if so, did you read them? Or did you read a paper that somehow tracks patient interest and simply forgot to cite it here?

**Response:** We gratefully appreciate your comments.

Q1: As has been noted by other Reviewers, the Discussion section is too long.

A: We have reorganized the discussion section to make the shortcomings of the different treatment options and suggestions for improving readability more concise.

Q2: However, on Page 19, you mention that patients’ interest level in CAM articles is due to including the topic of nutritional therapy and mentioning “commonly used foods, such as oranges and spinach”. Where is the citation for that? How did you determine interest? Does WeChat display the number of reads for a WPA? Even if you had such usage data, how can you determine which aspect of the paper engendered interest? Does WeChat allow users to post comments, and, if so, did you read them? Or did you read a paper that somehow tracks patient interest and simply forgot to cite it here?

A: We determined that patients were interested in nutrition therapy for CAM articles according to the reference [46].The researchers investigated 1686 articles on "Breast cancer Mutual support circle" published in the WPA and tracked the reading feedback of 148 WPA users. Because the articles in the WPA allow users to like, comment, collect, forward and share, so the researchers inferred users' interests based on the reads, retweets and feedback from users. The results showed that the articles focusing on nutritional therapy and postoperative behavior guidance were more liked and "impressed" by the users. Please see the revised content on page 16, line 321-324.

3. A similar question is raised in the next paragraph where you state,” medical treatment articles tended to lack descriptions of behavioral information that patients valued”. Again, how do you determine what patients value or what they like? Scoring papers on readability or suitability is logical, based on objective, known assessment scales, but determining reasons for popularity is not.

**Response:** Thank you for your constructive comment. The original expression was not appropriate and has been revised. See page 17, line 345-352.

4. On Page 21 you discuss a circular “Clinical value-oriented clinical development guidelines for antineoplastic drugs” issued by the Chinese Drug Review Center of the State Drug Administration. Is this a WPA? If so, was this written as a WPA, or was it simply posted to WeChat? This passage does a thorough job of articulating the perceived deficiencies of the circular (and again, we are told what patients want with no context or citation, “clinical trial articles provided little information about behavioral skills and applications of the information described, which are precisely what patients are most interested in and want to read”), but if this circular isn’t actually a WPA is this relevant?

**Response:** Thank you for the detailed review.

Q1: A circular “Clinical value-oriented clinical development guidelines for antineoplastic drugs” issued by the Chinese Drug Review Center of the State Drug Administration. Is this a WPA? If so, was this written as a WPA, or was it simply posted to WeChat?

A: The Guidelines for “Clinical value-oriented clinical development guidelines for antineoplastic drugs” is an official implementation guideline for clinical trials, which is published on the organization's official website and not on WPA. We mentioned this guideline here was to explain why the clinical trials was evaluated as one of the categories of treatment options in this paper.

Q2: This passage does a thorough job of articulating the perceived deficiencies of the circular (and again, we are told what patients want with no context or citation, “clinical trial articles provided little information about behavioral skills and applications of the information described, which are precisely what patients are most interested in and want to read”).

A: We've added references[34], and the revised content was on page 19, line 379-381.

Q3: If this circular isn’t actually a WPA, is this relevant?

A: Please see the answer to Q1 above.

5. My final question has to do with what you hope to achieve with this study, or, perhaps more importantly, how you plan on carrying out this plan. On Page 11 you state, “The present study, therefore, assessed the readability of information available in WPAs regarding different treatment options for breast cancer to clarify the dimensions of relatively high and low readability of each type of information, and to help WPA producers focus on improving the readability of this information”. This is an admirable goal – the world is awash in misinformation, especially on social media platforms discussing medical topics. What is your plan for sharing this study with WeChat? Do you plan on sending the Editorial Board a link to the final, published paper? Are you hoping that WeChat content creators will come across this paper on their own? As a peer reviewer I was taught to always ask a final question about generalizability or ease of implementation, and I don’t know you intend to transmit your findings to your intended audience so that your recommendations can be implemented.

**Response:** Thanks for bringing up the concern. Firstly, we hope that this article will be read by the researchers and practitioners in the field of health information communication or health education. They will apply the findings to their practice or future researches. Secondly, we can submit the related articles to the WPAs that focused on breast cancer treatment and spread our findings or suggestions through these platforms. Finally, we will participate in some academic conferences to disseminate this study to article producers or publishers who are providing medical care for breast cancer patients. So we believe that if the paper can be published, the recommendations will be implemented in future.

Once again, we appreciate you for your time and effort on revision, and the constructive comments and advice that improve out manuscript.

If the reviewers and editors have questions or are not satisfied with our revisions and responses, please let us know. We are willing to actively modify the text to improve the quality of the article.
