# Supplementary material for: Comparative readability of information on different treatment options for breast cancer, based on WeChat public accounts
Source: PLoS One. 2025 Jan 24;20(1):e0317032. doi: 10.1371/journal.pone.0317032 (PMC11759364; doi:10.1371/journal.pone.0317032)
Supplement: Attachment — Submitted filename: review.docx [file pone.0317032.s007.docx]

The SAM tool has been utilized in measuring the readable level of breast cancer treatment information that can be found on WeChat. Through the use of CAM articles, the readers got to see that the readability was higher in comparison to those in medicine and surgery. The clinical trials were disapproved of as the weakest part of the content and graphics. The authors advise the usage of more images and other related interactive elements to exhort the right readers to read the articles.

It is a good article with a clear structure and the correct order of the sections. The article was easy to the eyes, and the points were well developed but the title is too long. The main problem is candidly communicated and the area of emphasis is chosen by an example.

The abstract is laid out skillfully and follows a clear sequence of thoughts. The introduction, firstly, provides the research context and its significance in a well-constructed manner, so the readers are sure of the urgency of the issue (readability of health information) informing the most recent statistics and data. Nevertheless, the introduction might be more focused and relay only the scientific implications of the study. The methods are unambiguously and completely explained with a perfect description of the sampling, the (SAM) instrument used as well as the statistical analysis.

The findings that are tabulated and charted in the best way to make them more comprehensible are exhibited. The discussion is a deep analysis of the results and elucidates on the points for the differences between the groups. In general, the article is scientific and systematically organized; even so, it might be improved with the following additions.

"Is the sole use of the SAM tool sufficient for assessing readability? Why have other multidimensional criteria, such as the use of language complexity models or natural language processing (NLP) analyses, not been employed to examine the complexity of sentences and language structures in these articles? Can multidimensional assessments provide more comprehensive results?"

"For the reason that of the study is confined to Chinese WeChat articles, how do we ensure the absence of cultural bias in the readability analysis."

"Have the educational level and prior knowledge of the patients been taken into consideration in the readability analysis of the articles? How do we make sure that the reviewed articles are suitable for a diverse audience with different health literacy levels?"

What ways can we propose to make the articles more comprehensible for the audiences with the lower literacy levels?

Taking into account the fact that a major issue highlighted in the analyzed articles is the lack of pictures and illustrations that go along with the written content, what types of visuals might be the ones to improve the clarity and the comprehension?
