# Supplementary material for: Comparative readability of information on different treatment options for breast cancer, based on WeChat public accounts
Source: PLoS One. 2025 Jan 24;20(1):e0317032. doi: 10.1371/journal.pone.0317032 (PMC11759364; doi:10.1371/journal.pone.0317032)
Supplement: Attachment — Submitted filename: Response to Reviewers.docx [file pone.0317032.s008.docx]

**Point-by-point response to reviewer**

Manuscript ID PONE-D-23-12580R1

Title: Comparative readability of information on different treatment options for breast cancer, based on WeChat public accounts

Dear editors and reviewers,

We appreciate the time and effort that you and the reviewer dedicated to our manuscript. We are also grateful for the insightful comments and valuable suggestions. In the revised manuscript, the changes are marked in the “Track Changes” mode. Below are notes on the revised manuscript, with our answers highlighted in blue. We hope that the revision has addressed the concerns raised by the reviewer, and the revision is now found satisfactory. We are ready to work on resolving any remaining issues.

Sincerely,

Bingyan Li

Corresponding author:

Lunfang Xie

E-mail: [527548725@qq.com](mailto:527548725@qq.com)

**Responses to Reviewer #5**

1. Articles on breast cancer treatment options found in WeChat are not necessarily the result of research by authors in their specialty, are not peer-reviewed, and have no real experimental data to offer. So these articles are not credible. There is no academic value in studying these articles.

**Response:** We gratefully appreciate your comments.

(1) National Health Commission of China encourages medical staff to use wechat official accounts and other new media to disseminate health knowledge to improve the public's health knowledge reserve. **Nurses, as an important part of medical staff, can provide the public with understandable and useful health information, which will play a role in improving their health literacy.** Studies have shown that health wechat public account has become an important way for breast cancer patients to obtain treatment information, but no study has evaluated and compared the readability level of different treatment types of breast cancer information in wechat official accounts. In view of this, health related professionals such as nursing staff should evaluate and understand the real level of readability of breast cancer treatment information in wechat public accounts, in order to provide a basis for designing and selecting highly readable breast cancer treatment information, and improve the effect of health education.

(2) This article is part of our study named “quality and readability evaluation on breast cancer treatment information on WPAs”, and anther paper about the quality of informaition has been published. Among the 37 WPAs, individual users accounted for the largest share (20, 54%). To ensure the authenticity and security of WPAs, Tencent provides authentication services for WPAs. The authentication information and the WeChat Authentication Unique Identity are displayed in the authentication details of the account. The median of overall quality of 136 articles was 44 (interquartile range = 10.75) and ranked as "**fair**", of which 28 (21%) were of "**good**" or higher quality. cf. [33].Yang W, Li B, Liu M, et al. Quality evaluation of health information about breast cancer treatment found on WeChat public accounts. Arch Public Health. 2023;81(1):170. Published 2023 Sep 15. In particular, DISCERN which consists of three parts and 16 items was used to assess quality of 136 articles. **The first part (Items 1–8) is related to the reliability of the article; the second part (Items 9–15) evaluates the specific details of treatment choice information;** the third part (Item 16) describes the overall quality of the source publication of health information on treatment choices. **The reliability dimension was assessed as fair**, with criteria including: clear information sources, explicit objectives, relevant content themes, objective content, and mention of uncertainties. Please see the revised content on page 5, line 107-109.

(3) In our study, two postgraduate medical students (BL and WY) used SAM to evaluate the readability of the articles. BL and WY are part of our research team who has been studying on the readability of health information in China in recent years. The following articles are the relevant achievements of our team in recent years.

[1] **Yang W**, **Li B**, **Xie L**, et al. Quality evaluation of health information about breast cancer treatment found on WeChat public accounts. Arch Public Health. 2023;81(1):170. Published 2023 Sep 15.

[2] Wang Q, **Xie L**, Xing L, et al. Development of the readability evaluation index system for online health information. Chinese Journal of Nursing Education 2022;19(01):14-20.

[3] Wang L, **Xie L**, Wang Q, et al. Readability assessment of online health information for Systemic Lupus Erythematosus. Nursing Research of China 2020;34(24):4344-4349.

[4] Wang L, **Xie L**, Wang Q, et al. Study Status and Prospect of Readability Assessment of Online Health Information. Journal of Medical Informatics 2020;41(12):20-25+40.

[5] ZhiWei W, **Xie L**, YuanYuan F, et al. The readability assessment of printed education materials on Hypertension. Chinese Journal of Health Education 2020;36(12):1122-1125+1167.

[6] Wang Q, **Xie L**, Li M, et al. Review on readability assessment tools for health education materials. Chinese Journal of Health Education 2019;35:66-71.

[7] Wang Q, **Xie L**, Li M, et al. Evaluation of quality and readability of health education materials in Anhui Province from nurses’perspectives. Chinese Journal of Modern Nursing 2019;25:1093-98.

[8] **Li B**, **Liu M**, Liu J, Zhang Y, Yang W, **Xie L**. Quality assessment of health science-related short videos on TikTok: A scoping review. Int J Med Inform. 2024 Jun;186:105426.

[9] **Liu M**, Zhou Y, **Li B.** Research progress of health science video quality evaluation tools. Chinese General Practice. DOI: 10.12114/j.issn.1007-9572.2023.0683.

Please see the revised content on page 8, line 173-174.

**Responses to Reviewer #7**

1. The SAM tool has been utilized in measuring the readable level of breast cancer treatment information that can be found on WeChat. Through the use of CAM articles, the readers got to see that the readability was higher in comparison to those in medicine and surgery. The clinical trials were disapproved of as the weakest part of the content and graphics. The authors advise the usage of more images and other related interactive elements to exhort the right readers to read the articles. It is a good article with a clear structure and the correct order of the sections. The article was easy to the eyes, and the points were well developed but the title is too long. The main problem is candidly communicated and the area of emphasis is chosen by an example.

**Response:** We gratefully appreciate your comments.

(1) The purpose of this study is to evaluate the readability of articles on different treatment methods of breast cancer in WPAs. We tried to modify the title, but it was found that the revised title did not reflect the research focus in detail, so the title is not modified for the time being after discussion by the research team. If the experts have good advice to offer, we will consider it carefully.
(2) In the discussion section, in order to fully reflect the application value of the results of this study, our team not only analyzed each dimension of SAM, but also analyzed each item and made personalized suggestions accordingly. Referring to the expert's parsimonious advice, we have only condensed the questions, but not the recommendations. Please see the revised content on page 16, line 307-311.
2. The abstract is laid out skillfully and follows a clear sequence of thoughts. The introduction, firstly, provides the research context and its significance in a well-constructed manner, so the readers are sure of the urgency of the issue (readability of health information) informing the most recent statistics and data. Nevertheless, the introduction might be more focused and relay only the scientific implications of the study. The methods are unambiguously and completely explained with a perfect description of the sampling, the (SAM) instrument used as well as the statistical analysis.

**Response:** Thank you for pointing this out. Based on your suggestion, the contents of the introduction have been briefly expressed, focusing on the research significance. Please see the revised content on page 4, line 73-84 and page 5, line 97-109 .

3. The findings that are tabulated and charted in the best way to make them more comprehensible are exhibited. The discussion is a deep analysis of the results and elucidates on the points for the differences between the groups. In general, the article is scientific and systematically organized; even so, it might be improved with the following additions. "Is the sole use of the SAM tool sufficient for assessing readability? Why have other multidimensional criteria, such as the use of language complexity models or natural language processing (NLP) analyses, not been employed to examine the complexity of sentences and language structures in these articles? Can multidimensional assessments provide more comprehensive results?"

**Response:** We gratefully appreciate for your comment. This study evaluated the readability of articles on different treatments of breast cancer in WPAs **from the perspective of non-text factors**, and therefore did not use the language complexity models or natural language processing (NLP) . On the other hand, there is a dimension of "literacy needs" in SAM, and the words and phrases of articles were also briefly evaluated. The assessment items included: (1) Writing style, active voice; (2) Vocabulary uses common words.

4. For the reason that of the study is confined to Chinese WeChat articles, how do we ensure the absence of cultural bias in the readability analysis.

**Response:** Thanks for bringing up the concern. The SAM was translated into Chinese by Li Xianwen in 2010^[1]^. Since then, the SAM scale has been widely used to evaluate the readability of health information in China, verifying the applicability of the scale in evaluating the readability of health information in China. In addition, after obtaining the authorization of the English version of SAM, **the research group compiled the SAM handbook from the perspective of Chinese users by referring to the Chinese version of SAM by Li Xianwen. Each item was explained in detail and examples were provided to avoid cultural bias due to possible differences in the understanding of each item between Chinese and foreigners.** In order to verify the comprehensibility of the SAM handbook and the accurate expression of SAM items, we evaluated the readability of SLE health information in 2020^[2]^. At the same time, the views of SLE patients with high and low health literacy on readability were collected through personal interviews to verify the applicability of the SAM handbook in evaluating the readability of health information in China. Therefore, the assessors of this study used the validated SAM handbook to assess the readability of breast cancer treatment information in the wpa to reduce cultural bias in the assessment process. Please see the revised content on page 8, line 173-174.

**References:**

[1] Xianwen Li. Suitability evaluation of the health education text material and the health literacy intervention of Korean-Chinese older adults with hypertension. [D] Yanjie: Yanbian University, 2013.

[2] Q Wang. Readability assessment of printed health education materials regarding Systemic Lupus Erythematosus[D].Anhui Medical University,2021.DOI:10.26921/d.cnki.ganyu.2020.001063.

5. "Have the educational level and prior knowledge of the patients been taken into consideration in the readability analysis of the articles? How do we make sure that the reviewed articles are suitable for a diverse audience with different health literacy levels?"

**Response:** Thank you for pointing this out.

The score of SAM was "superior", indicating that the health information could be understood by the target population with low health literacy^[1]^. When SAM score is "adequate", it means that health information can be understood by the target population with high health literacy^[2]^. The SAM score of "not suitable" indicates that the health information cannot be understood by the target population^[3]^.

There is no assessment of education level and prior knowledge involved in the impression, and the traditional assessment results are understandable to most people. However, no research reports correspond to specific grades, unless the English readability assessment formula is used, which is only evaluated from the perspective of words, without considering other important dimensions, and the evaluation of readability is not comprehensive enough.

**References:**

[1]Tzeng YF, Gau BS. Suitability of asthma education materials for school-age children: Implications for health literacy. J Clin Nurs. 2018 Mar;27(5-6):e921-e930.

[2]Taylor-Clarke K, Henry-Okafor Q, Murphy C, Keyes M, Rothman R, Churchwell A, Mensah GA, Sawyer D, Sampson UK. Assessment of commonly available education materials in heart failure clinics. J Cardiovasc Nurs. 2012 Nov-Dec;27(6):485-94.

[3]Martin CA, Khan S, Lee R, Do AT, Sridhar J, Crowell EL, Bowden EC. Readability and Suitability of Online Patient Education Materials for Glaucoma. Ophthalmol Glaucoma. 2022 Sep-Oct;5(5):525-530.

6. What ways can we propose to make the articles more comprehensible for the audiences with the lower literacy levels?

**Response:** Thank you for your constructive comment. When the SAM score is "superior", it means that the health information can be understood by the target population with low health literacy. When the SAM score is "adequate" or "not suitable", it means that the health information cannot be understood by the target population with low health literacy. Therefore, when the SAM score is "adequate" or "not suitable", the low literacy target population must be valued. In this study, the SAM scale was used to evaluate and compare the readability of articles on different treatment methods for breast cancer in WPAs, and the results showed that the overall readability was "adequate". Suggestions were made based on items with low SAM scores, see page 16, line 307-311 for details. Recommendations for medical and comprehensive care articles are provided on page 17 and 18, line 329-339. "We also subcategorized articles on medical treatment and CAM treatment, evaluated and compared their readability, and made recommendations for improvement based on readability results, as detailed on page 18, line 356-366 and page 19, 373-376."

7. Taking into account the fact that a major issue highlighted in the analyzed articles is the lack of pictures and illustrations that go along with the written content, what types of visuals might be the ones to improve the clarity and the comprehension?

**Response:** Thanks for bringing up the concern.The "graphic" dimension of the SAM scale focuses on the illustration of the article, also known as the pictures. Combining illustration and text can improve the readability of information. Here are some examples and applications of illustrations.

(1) Explanatory illustration: its role is to assist the text, to achieve a visual effect. For example, some health information will involve some acupoints and various movements of the human body. It is necessary to draw some "illustrations" to assist the explanation, so that the audience can see the specific location at a glance.
(2) Embellished illustration: their role is to liven up the page and fill in the blanks. This kind of illustration is used to modify the layout effect. No strict text content requirements, flexibility is strong. It can be used to beautify the title, fill in the gaps where the text is not enough, and can also be used to distinguish the structure of the text block.
(3) Thematic illustration: its role is to deepen the theme, show the theme, publicize the theme. Health information may change with the change of season and epidemic season, so each issue has its theme, and each page has its theme content. Therefore, the illustrations on the layout should also be conceived and expressed according to different themes. Such illustrations can not only serve as a foil, serve between the words, but also can be independent into paintings, creating a distinct feeling effect. Present a theme with text on the same page.

Once again, we appreciate you for your time and effort on revision, and the constructive comments and advice that improve out manuscript.

If the reviewers and editors have questions or are not satisfied with our revisions and responses, please let us know. We are willing to actively modify the text to improve the quality of the article.
